# Spatial and Genetic Epidemiology of Hookworm in a Rural Community in Uganda

**DOI:** 10.1371/journal.pntd.0000713

**Published:** 2010-06-15

**Authors:** Rachel L. Pullan, Narcis B. Kabatereine, Rupert J. Quinnell, Simon Brooker

**Affiliations:** 1 Department of Infectious and Tropical Diseases, London School of Hygiene and Tropical Medicine, London, United Kingdom; 2 Vector Control Division, Ministry of Health, Kampala, Uganda; 3 Institute of Integrative and Comparative Biology, University of Leeds, Leeds, United Kingdom; 4 Malaria Public Health and Epidemiology Group, Kenya Medical Research Institute-Wellcome Trust Research Programme, Nairobi, Kenya; George Washington University, United States of America

## Abstract

There are remarkably few contemporary, population-based studies of intestinal nematode infection for sub-Saharan Africa. This paper presents a comprehensive epidemiological analysis of hookworm infection intensity in a rural Ugandan community. Demographic, kinship, socioeconomic and environmental data were collected for 1,803 individuals aged six months to 85 years in 341 households in a cross-sectional community survey. Hookworm infection was assessed by faecal egg count. Spatial variation in the intensity of infection was assessed using a Bayesian negative binomial spatial regression model and the proportion of variation explained by host additive genetics (heritability) and common domestic environment was estimated using genetic variance component analysis. Overall, the prevalence of hookworm was 39.3%, with the majority of infections (87.7%) of light intensity (≤1000 eggs per gram faeces). Intensity was higher among older individuals and was associated with treatment history with anthelmintics, walking barefoot outside the home, living in a household with a mud floor and education level of the household head. Infection intensity also exhibited significant household and spatial clustering: the range of spatial correlation was estimated to be 82 m and was reduced by a half over a distance of 19 m. Heritability of hookworm egg count was 11.2%, whilst the percentage of variance explained by unidentified domestic effects was 17.8%. In conclusion, we suggest that host genetic relatedness is not a major determinant of infection intensity in this community, with exposure-related factors playing a greater role.

## Author Summary

Detailed descriptions of the epidemiology of intestinal nematode infections within affected communities remain of considerable importance for the effective design of disease control programmes. We therefore conducted a parasitological survey of 1,803 individuals living in a rural community in eastern Uganda. Two complementary analytical approaches were used to evaluate causes of variation in intensity of hookworm infection in this community, both of which exploit correlation structures between individuals. Infection intensity was higher among older individuals and was also associated with factors influencing environmental exposure to infection, socio-economic indicators and treatment history. After accounting for these risk factors, spatial correlation remained evident between households less than 82m apart. Focusing further on similarities within households, our results suggest that 11% of variation in infection intensity could be explained by genetic differences between individuals and 18% by unmeasured factors associated with the domestic environment. Taken together, these results suggest that exposure-related factors, rather than host genetics, have the greatest influence on variation in infection intensities.

## Introduction

Recent years have seen an unprecedented expansion in financial and technical support for school-based deworming, with an increasing number of countries in sub-Saharan Africa implementing nationwide control. The initiation of such control does not however signal the end of epidemiological research and detailed data on patterns and risk factors for infection are still required for the refinement of ongoing control activities. It is surprising therefore that there are so few population-based studies of the epidemiology of hookworm in Africa. Those that do exist typically describe age-related changes in infection prevalence and intensity, demonstrating consistent increases with age, peaking in adults [1]–[6], pronounced aggregation of high intensity infection within high risk individuals [1], , and villages [5], [6], and providing evidence for predisposition to low or high intensity infection [1] using faecal egg counts as a proxy; only one study has looked at actual worm burdens [7]. Remarkably few investigations have been able to address spatial and/or genetic determinants of infection within African communities. However, our ability to investigate such factors today has been greatly facilitated by the increasing availability of high resolution spatial data and the advent of powerful analytical tools, including Geographic Information Systems (GIS), readily implemented Bayesian analysis and quantitative genetic approaches [8]–[12]. For example, GIS-based studies in a cohort of South African primary school children have demonstrated considerable spatial clustering of infection within a smaller area, strongly influenced by several environmental factors [10]. Similarly, several studies outside Africa have reported a significant role for both host genetics and the family environment in determining hookworm infection intensity [12]–[14], although the only genetic epidemiology investigation of hookworm in Africa did not account for the effects of shared family environment [15].

This paper reports results from a population-based study of hookworm infection in Uganda. Our aim is to provide a detailed description of hookworm epidemiology for sub-Saharan Africa. In order to comprehensively investigate determinants of infection intensity two complementary analytical approaches are employed. First, negative binomial spatial modelling is used to investigate spatial variation in the intensity of infection, whilst adjusting for individual- and household-level covariates. Second, genetic variance component analysis, a quantitative genetic approach which takes into account familial relationships within and between households, is used to determine the relative contributions of host genetics (and other factors) to variation in infection intensity.

## Methods

### Study area and population

The study was conducted in 2008 in four villages in Mulanda sub-county, which is located in Tororo district, eastern Uganda. The area is characterised by dry savannah grassland interrupted by bare rocky outcrops and lower lying swamps, with an average daytime temperature of 27°C and two rainy seasons (March to May and August to October). The majority of inhabitants are involved in rural subsistence farming, with garden plots in very close proximity to farmers' compounds. Houses are predominantly of traditional construction (mud walls, thatched roofs), approximately 95% of households have a pit latrine and residents collect their water from boreholes or local protected springs. Since 2004, periodic mass chemotherapy in schools has been conducted on three occasions as part of Child Health Days Plus, although the coverage has been variable, ranging from 30 to 65%; no community-based treatment has been implemented.

### Census and recruitment

Between June and August 2008, all households in the sub-county were visited for census enumeration, and a standard questionnaire was used to record the demographics of each household and information on asset ownership, crowding, water and sanitation and construction. Information on ownership of household assets was used to construct a wealth index for each household using principal component analysis (PCA), as described in [16]. The resulting score was divided into quintiles, to provide a categorical measure of relative socio-economic status. Household locations, health care facilities and major infrastructure were mapped using a hand-held eTrex global positioning system (Garmin Ltd., Olathe, KS). Geographic data were compiled and maps created using ArcGIS 9.2 (Environmental Systems Research Institute Inc., Redlands, CA, USA). Subsequently, four representative contiguous villages were purposively selected on the basis of size and accessibility, and all households in these villages re-visited between September and December 2008. Adults and parents/guardians of children had the purpose of the study explained to them in the language they felt most comfortable with and were asked if they wished to participate. All willing residents aged over 6 months that were resident in the study site over the last 24 months, could be unambiguously tied to a single household, and did not attend school/work full time outside Mulanda parish, were eligible to participate. Excluded individuals were still offered parasitological examinations and treatment, but were not considered part of the data set for analysis. Signed informed consent was obtained from all adults and parents/guardians willing to participate and written assent from children aged 13–18 years. The study protocol was approved by the Makerere University Faculty of Medicine Research and Ethics Committee (#2008-043) Uganda National Council of Science and Technology (#HS 476) and London School of Hygiene and Tropical Medicine Ethics Committee (#5261).

### Procedures

Participants were asked to provide two daily consecutive stool samples (26.3% of participants provided only one sample) which were examined in duplicate by the Kato-Katz technique (using 41.7 mg slides) within 45 minutes of preparation and infection intensities expressed as eggs per gram (epg) of faeces using the arithmetic mean of all slides. Infected individuals were treated with a single 400 mg dose of albendazole. A standardized questionnaire was administered to adults and to primary carers of children to record details of previous anthelmintic treatment (user reported), education-level, school attendance and occupation and protective behaviours. Household heads were also interviewed about relationships between all household members, and about first- or second-degree relatives living in other households in the study villages, thereby identifying genetic links within and between households. Individuals were then defined as belonging to the same extended pedigree if they were related to anyone else in the pedigree or were married to anyone in the pedigree. Absent parents or family members connecting residents were assigned identification numbers and missing phenotype data. Pedigrees were assembled and indexed using PEDSYS [17] and visualised using Cranefoot [17], and any doubtful pedigree relationships were confirmed by re-interviewing selected household members.

### Analysis overview

**Figure 1:**
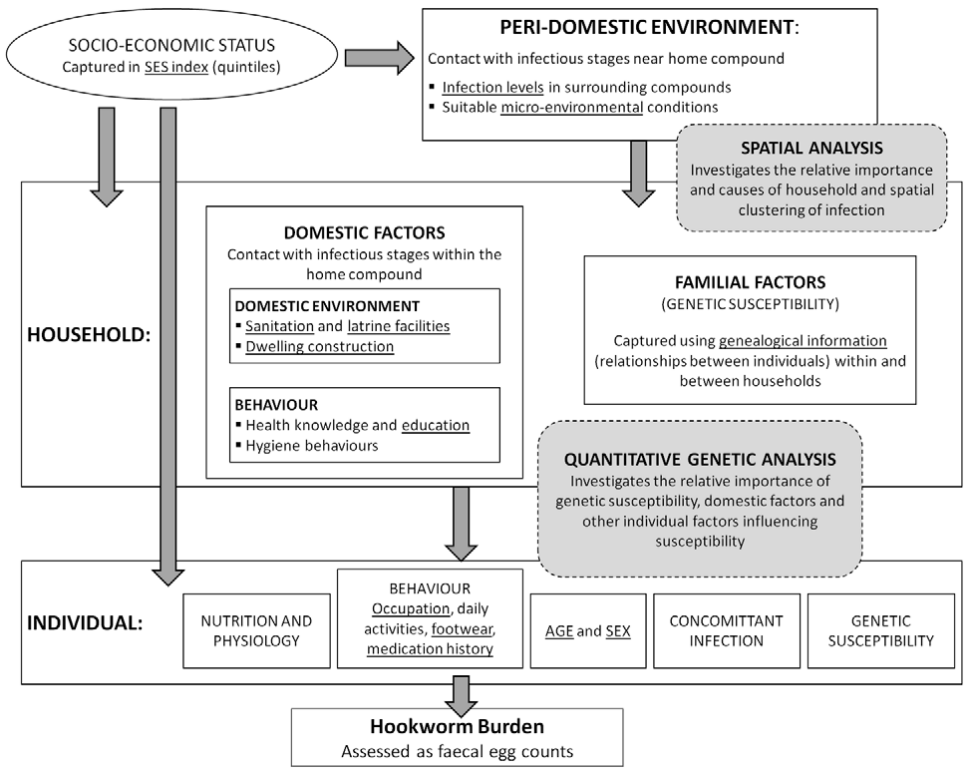
Representation of the relationships between peri-domestic, household and individual variables and hookworm burden. Includes the areas of this framework addressed by complementary analysis strategies used in this paper. For example, socio-economic factors (assessed, for example, by asset ownership) act through a number of inter-related proximate determinants, which include environmental factors (such as availability of water and sanitation), behavioural factors (such as health seeking behaviours) and nutrition (including anthropometric status and diet) which in turn may affect the risk of an individual being infected with hookworm. Covariates with available data are underlined. Both analyses address hookworm intensity (epg): the spatial analysis uses a negative binomial model, incorporating an over dispersion parameter (k) to account for extra-Poisson variation; quantitative genetic analysis (genetic variance component analysis) uses a linear regression model, therefore data was log-transformed prior to analysis. These methods both exploit correlation structures in egg counts: for example, if there is a genetic basis to susceptibility, correlation due to genetic effects may be expected to decrease with distance in the pedigree, whereas if exposure to infective hookworm larvae most commonly occurs outside the household (for example in agricultural areas or defined defecation sites away from the household [42]), correlation due to environmental effects may decrease slowly with physical distance between households.

The transmission dynamics of soil-transmitted helminth infection, including hookworm, are primarily determined by the number of worms present in the host (infection intensity), rather than the number of hosts infected [18]; analysis therefore focused on quantitative egg counts (intensity of infection),an indirect measure of worm burden. Data analysis was guided by a conceptual framework which takes account of the hierarchical relationships between peri-domestic, household and individual factors (Figure 1). In this framework, we utilise two complementary analytical approaches which both exploit correlation structures between individuals to evaluate aggregation in egg counts according to different sets of relationship, namely spatial and genetic relationships. Independent households within the same family compound shared the same location and peri-domiciliary environment and as such were treated as single units for both spatial and genetic analysis. Thus, in this report “household” refers to a family compound. Spatial analysis adopted a Bayesian geo-statistical approach incorporating both spatial correlation and non-spatial clustering at a household-level, whilst accounting for included covariates. Analysis of genetic factors was based on genetic variance component analysis whereby the relative importance of genetic susceptibility, other domestic factors and (non-genetic) individual factors in determining infection intensity was assessed. This was done via a frequentist variance partition approach, using degrees of relationship within and between households in order to partition variation into its genetic, domestic and other causes.

### Preliminary analysis

Egg counts are usually over-dispersed, and as such are often described well by the negative binomial distribution [19]. Negative binomial regression models were therefore applied to investigate factors associated with the intensity of hookworm infection. These models include an over-dispersion parameter, *k*>0, which incorporates extra-Poisson variation. Preliminary investigation of covariates followed a frequentist approach, with standard errors adjusted for non-independence of individuals within households using robust Huber/White/Sandwich variance estimates, using Stata version 10 (Stata Corporation, College Station, Texas). Explanatory variables significant at the 10% significance level were entered into the multivariate negative binomial regression models, and backwards-stepwise elimination was used to generate a minimum adequate model; excluded covariates (P>0.05) were retested in the minimal model.

Quantitative genetic analysis, however, relies on linear regression, which assumes the outcome to be normally distributed. The data were therefore log-transformed, and multivariate linear regression models developed following the same stepwise procedures described above.

### Bayesian negative binomial spatial models

Spatial variation in infection intensity (and spatial clustering, if evident) was investigated by fitting a negative binomial distribution to the total egg count using the Bayesian spatial model of Alexander et al (2000), assuming a spatially constant *k* and including grams of faeces examined as an offset. Models were developed in WinBUGS Version 14. Between-household (non-spatial) variation was accounted for by a household-level random effect, *u_j_*, with an exchangeable correlation matrix (mean 0, variance σ_1_^2^). Fixed effect parameters from the negative binomial regression models are presented as density ratios (DR), that is, exp(β) where β is the vector of regression coefficients. For ease of interpretation, 100*(DR-1) provides the estimated percentage chance in egg counts for each unit increase in the independent variable. The spatial random effect, *v_j_*, was modelled as a stationary Gaussian process with a mean 0, variance σ_2_^2^ and correlation function exp(−*α d_kl_*), where *d_kl_* is the straight-line distance between households *k* and *l*. The geographical variability, σ_2_^2^, represents the spatially constructed component of variability on a log scale; the exponential of this parameter, exp(*v_j_*), yields a ratio by which the house's mean egg count is higher or lower than expected, which can be thought of as a standardised parasite ratio (SPR) [11]. The smoothing parameter *α* measures the rate at which the spatial correlation decays to zero with increasing distance; ln(2*α*) is the ‘half-distance’ (i.e. the distance over which the correlation decays by half), and 3*α* is the distance at which the correlation reduces by 95%. Thus, the modelled mean egg counts and household clustering incorporate a degree of smoothing, which is dependent on the fitted spatial correlation.

Fixed regression coefficients were assigned diffuse priors functionally equivalent to a vague normal prior with mean 0 and large variance; spatial and non-spatial variance components (σ_1_^2^ and σ_2_^2^) were assigned gamma distributions; the over-dispersion parameter *k* was also assigned a gamma distribution with density proportional to *k* ^(0.5)^ exp(−0·01*k*) so that, *a priori*, *k* had a high variance; a weakly-informative uniform prior was assigned to *α*, with parameters defined by semivariograms estimated on the basis of logarithmically transformed count data (i.e. ln(epg+1)), averaged by household, using the R module *GeoR*. We ran 35,000 iterations, with the first 10,000 discarded (‘burn in’) and sub-sampled every 5^th^ observation, giving a final sample size of 5000 for which model parameters were estimated. Convergence was evaluated on the basis of inspection of sample traces, and inference was based on the better fitting model using the deviance information criteria (DIC) as a goodness of fit measure.

### Quantitative genetic analysis

Heritability estimation was undertaken using genetic variance component analysis implemented in SOLAR 4.2.0 (Southwest Foundation for Biomedical Research, Texas) [20]. A series of mixed linear regression models were fitted to logarithmically-transformed arithmetic mean egg counts, i.e. ln(epg+1), using maximum likelihood procedures. Residual kurtosis for this trait was within acceptable range (i.e. <0.8). Additive genetic effects were separated from household and other individual effects through incorporation of a kinship coefficient matrix, thus allowing the covariance between relatives to depend upon the degree of relatedness between them [21], [22]. Chi-squared testing based on likelihood ratios was used to compare nested models and test the significance of variance parameters. Contributions of additive genetics (heritability, *h* ^2^) and household (*c^2^*) were standardised by dividing by the total phenotypic variation. The relative contribution of fixed covariates to total phenotypic variance was estimated by comparing the trait standard deviation in models with and without fixed covariates.

## Results

Complete questionnaire and parasitological data were available for 1,803 individuals (76.5% total population) and pedigree information for 1,687 (71.5%) individuals. There were 341 compounds, with 1–19 (median 5) phenotyped individuals per compound; an average compound comprised 2.9 phenotyped children (0–15 years). Comparison of census data for those that participated and those that chose not to participate suggests that our final study sample under- sampled adult males (*p*<0.001). There were however no statistical difference in the size or relative socio-economic status of participant and non-participant households [23]. Baseline characteristics of the study population are shown in Table 1.

**Table 1:** Baseline characteristics of study participants by demographic group.

Personal characteristics:	Number (%) stratified by demographic group		
	<5 years; n = 385	5–15 years; n = 659	≥16 years; n = 831
Hookworm infection:			
Prevalence, % (n/total)	26.5%	33.2%	51.5%
Geometric mean egg count, epg	82	87	196
Sex: male	187 (48.6%)	323 (51.3%)	300 (38.1%)
Anthelmintic in previous 6 months	137 (35.9%)	423 (67.5%)	141 (17.9%)
Wears shoes outside home	110 (28.6%)	101 (15.3%)	457 (58.0%)
Primary carer with any education	285 (74.4%)	437 (69.8%)	-
Currently attends school	-	537 (86.3%)	-
Ever attended school	-	-	432 (54.8%)
Occupation:			
Farmer	-	-	324 (41.1%)
Formal employment	-	-	58 (7.4%)
Student	-	-	55 (7.0%)
None	-	-	330 (41.9%)

Household characteristics:	Number (%) n = 435
Toilet facilities:	
Covered pit/VIP^a^	88 (20.2%)
Uncovered pit	326 (74.9%)
None	21 (4.8%)
Floor type:	
Mud/beaten earth	392 (90.1%)
Cement	43 (9.9%)
Member with formal income	63 (14.5%)
Educated household head^b^	29 (6.6%)

a VIP; ventilated improved pit latrine.

b Household head with education above secondary (higher education or vocational training).

**Figure 2:**
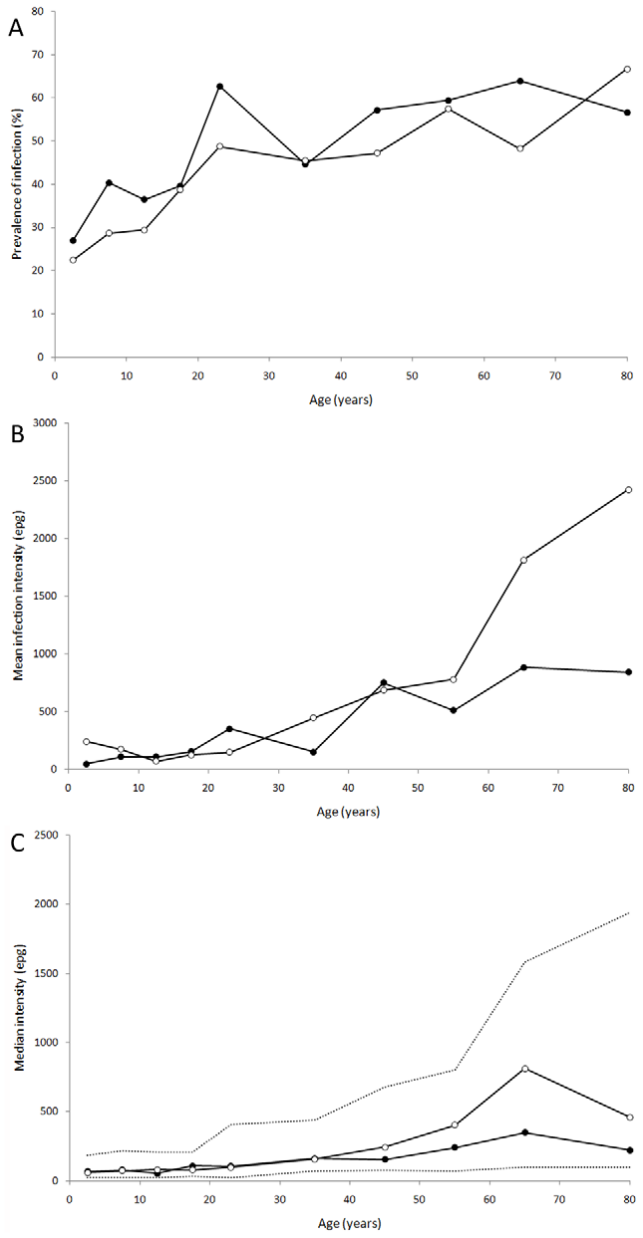
Age-sex distributions for hookworm infection. (A) prevalence of hookworm infection, (B) mean intensity of infection, in epg, and (C) median intensity of infection (in epg) for those infected only (dashed lines indicate the population interquartile range). Males, closed circles; Females, open circles.

The prevalence of hookworm was 39.3%, with the majority of infections (87.7%) ≤1000 epg. The geometric mean intensity of infection was 134 epg (95% confidence intervals [CI]: 118–153). Other helminth species were rare: prevalence of *Schistosoma mansoni* was 1.7%, *Trichuris trichiura* 0.2% and *Ascaris lumbricoides* 0.2%. The prevalence of hookworm did not differ significantly by sex. Prevalence rose steadily with age (Figure 2A), whilst intensity increased substantially from ∼35 years. Mean intensity among over 60 year olds was 2,318 epg (95% CI 864 to 3,773) in females and 936 (95% CI 387 to 1,485) in males (Figure 2B). However, much of this increase is due to very high egg counts in a few older individuals, and the increase in median intensity (in those infected) among the elderly was less marked (Figure 2C).

### Risk factors

Table 2 presents the results of the spatial model for hookworm intensity. In multivariate analysis there was no evidence of a difference between males and females, although intensity did rise with age. As expected, intensity was associated with factors influencing environmental exposure to infection, as well as socio-economic indicators and treatment history. Specifically, intensity was higher among older individuals and individuals who reported not receiving anthelmintic treatment in the previous six months, walking barefoot outside the home, with living in a household with a mud floor and with low education of household head. Intensity was also higher among individuals who provided two stool samples, a likely reflection of the increased likelihood of detecting an infection in this group.

**Table 2:** Final estimates for covariates and variance parameters for hookworm infection including variance parameter estimates.

	(i) spatial negative binomial model		(ii) genetic vca linear regression model	
covariates	Estimate	(95% BCI)	Estimate	(95% CI)
Individual characteristics:				
Age (per year)	1.08	(1.08,1.12)	1.04	(1.03,1.05)
Two stool samples (vs. one)	1.45	(0.83,2.27)	1.62	(1.23,2.12)
Barefoot (vs. shoes or sandals)	2.86	(1.71,4.51)	1.43	(1.12,1.84)
Anthelmintic within 6 months	0.29	(0.17,0.46)	0.51	(0.39,0.66)
Socio-economic indicators:				
Educated primary carer^1^	0.14	(0.04,0.37)	0.61	(0.44,0.84)
Educated household head^2^	-		0.66	(0.31,1.22)
Formal income	-		0.67	(0.44,1.03)
Household characteristics:				
Beaten earth flooring	20.99	(7.63,47.71)	3.03	(1.72,5.37)
>500m from rocky area	-		1.58	(1.14,2.20)
**random effect parameters (spatial negative binomial model):**				
*k* (over-dispersion parameter)	0.07	(0.0005)		
σ_1_ 2 (non-spatial household variation)	0.31	(0.04,1.05)		
σ_2_ 2 (spatial household variation)	1.31	(0.03,2.67)		
α (smoothing parameter)	27.23	(5.6,173.4)		
**variance components as proportions of residual variation (se) (genetic vca model):**				
Additive genetic			0.11	(0.06)
Shared domestic environment			0.18	(0.04)
Individual-specific			0.71	(0.05)

1 Household head with education above secondary.

2 Primary carer with any level of education (primary incomplete and above).

(i) Bayesian negative binomial geospatial model used to estimate the scale and importance of residual spatial correlation in mean household prevalence. The estimate of the negative binomial regression covariate parameters represent density ratio (DR) of egg counts. (ii) Genetic variance component analysis used to estimate the proportion of residual variance attributable to additive genetics, individual (non-genetic) and shared household effects. The estimate of the genetic VCA covariate parameters have been transformed back to the original egg count scale (exponentiated regression coefficients), and are thus multiplicative and directly comparable with the negative binomial DRs. It is not possible to standardise variance parameters in the spatial negative binomial model.

The estimate of the negative binomial regression coefficient parameters represent the density ratio (DR) of egg counts, and that of the genetic VCA linear regression model the exponentiated regression coefficients. Both are multiplicative.

### Residual spatial clustering

**Figure 3:**
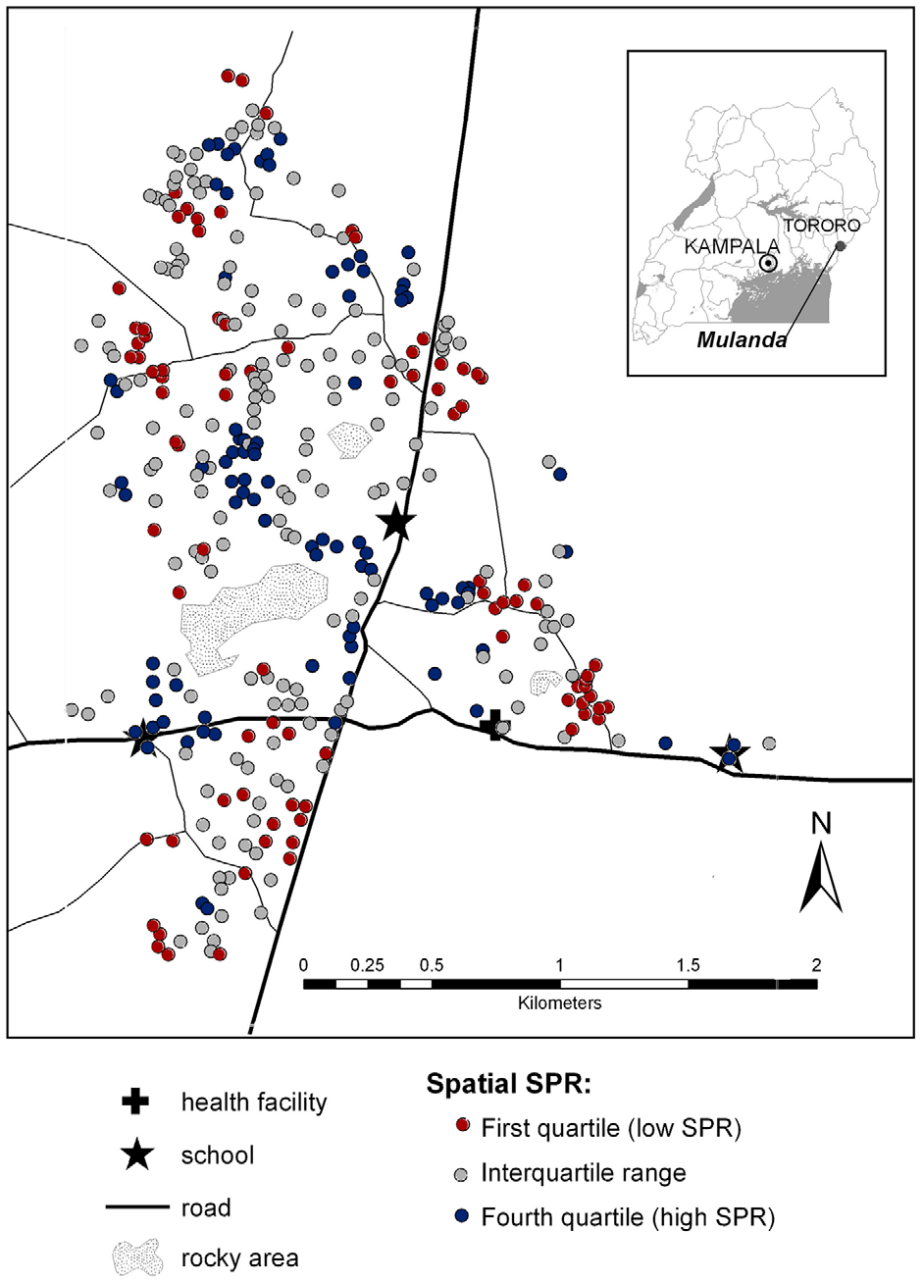
Map of the small-scale spatial heterogeneity of hookworm intensity in Mulanda, after adjusting for individual and household risk factors. Each dot represents a compound, with the shading showing the quartile of the standardised parasite ratio (SPR; ratio by which the compound's mean log egg count is higher or lower than expected) as derived by the Bayesian spatial model. High infection intensities are seen in the south-west, north-west and east of the study site, whilst low intensity infections are seen in the central region. Inset: location of the study site in eastern Uganda.

Intensity of infection exhibited significant household and spatial clustering, as indicated by the improved fit of models including household-level spatial and non-spatial random effects. Of the total variation between households, 81% could be explained by small-scale spatial clustering and 19% by non-spatial household clustering. The range of spatial correlation was estimated to be 82 m and was reduced by a half over a distance of 19 m. Figure 3 shows the model-based estimates of spatial variation in infection intensity and highlights three separate areas of high infection intensity.

### Household and genetic determinants of infection

Genetic variance component models were used to define the contribution of hereditary and environmental influences on intensity of infection, thereby providing an estimate of the total cumulative effect of individual genes (the “additive genetic” effect, or heritability) on overall variation. Of the 1,687 people for whom relationship information was available, 1,624 could be grouped into 155 pedigrees, with two large multi-generational pedigree comprising 690 and 173 phenotyped individuals respectively, 21 smaller pedigrees of 10–60 phenotyped individuals and 69 nuclear families (<10 members); 63 participants had no phenotyped relatives in the study area. When considering up to 8 degrees of relatedness (over 5 generations), data was available for 11,509 informative relative pairs; this includes 2,627 first degree relatives (e.g. siblings), 1,968 second degree relatives (e.g. grandparent/grandchild) and 2,291 third degree relatives (e.g. cousins).

Variance parameter estimates and corresponding model log-likelihoods are provided in Table 3. Intensity was best described by a saturated model which included both additive genetic and shared household effects. As shown in Table 3, risk factors identified as significantly associated with log-transformed infection intensity are consistent with results from the above spatial negative binomial model. When adjusting for these covariates (which explained 18% of total variation in log-transformed egg counts), heritability was estimated as 11.2% (Standard error [SE] 5.7%), with a further 17.8% of residual variation (SE 3.7%) attributable to other unmeasured domestic factors; 71% of residual variation (58% of total variation) remained unexplained by all factors in this model.

**Table 3:** Variance component analysis of heritability (*h* ^2^) and household effects (*c* ^2^) of intensity of hookworm infection.

		variance component estimates					
		Hookworm ln(egp+1)					
models		Ln*L*	*h^2^*	*c^2^*	*e^2^*	LR test	*P*
**no covariates**							
1	Sporadic (*e^2^*)	−2444			1		
2	Polygenic (*h^2^* + *e^2^*)	−2367	0.380		0.620	A (2 vs. 1)	<0.001
3	Household (*c^2^* + *e^2^*)	−2387		0.275	0.725	B (3 vs. 1)	<0.001
4	Saturated (*h^2^* + *c^2^* + *e^2^*)	−2364	0.106	0.235	0.659	C (4 vs. 3)	0.02
						D (4 vs. 2)	<0.001
**with covariates** +							
1	Sporadic (*e^2^*)	−2251			1		
2	Polygenic (*h^2^* + *e^2^*)	−2196	0.337		0.663	A (2 vs. 1)	<0.001
3	Household (*c^2^* + *e^2^*)	−2207		0.221	0.779	B (3 vs. 1)	<0.001
4	Saturated (*h^2^* + *c^2^* + *e^2^*)	−2194	0.112	0.178	0.710	C (4 vs. 3)	0.02
						D (4 vs. 2)	<0.001

**+:** Model adjusted for age (linear term), medication history, number of stool samples examined, footwear, household income, education level and residential location.

Chi-squared test of nested models; A: *h^2^*≠0 (not controlling for *c* ^2^) B:c*^2^*≠0 (not controlling for h^2^); C: *h^2^*≠0 (after controlling for *c* ^2^); D: c*^2^*≠0 (after controlling for h^2^).

## Discussion

Marked heterogeneity within communities has been long recognised for intestinal nematode infections [1], [10]–[12], [15], [18], [24]–[26], although there have been few studies undertaken in post-treatment settings in sub-Saharan Africa. This analysis sought to comprehensively assess major sources of heterogeneity (spatial, genetic and household) in the intensity of hookworm infection in a Ugandan population using both spatial and quantitative genetic modelling approaches. In keeping with previous studies [27]–[30] the highest infection intensities were found among older residents, and we identified a number of individual and household risk factors. After taking these features into account, we revealed substantial spatial clustering of infection intensity in this community, with the relatively small spatial range (81m) suggesting that much of the exposure to infection is concentrated in the peri-domiciliary environment. Focusing further on the household/family level, we estimate that 11% of residual variability in egg counts could be attributed to genetic differences between individuals (heritability) after accounting for individual and household risk factors. Unmeasured factors associated with the domestic environment explained a further 18% of residual variation. Results from quantitative genetic analysis also demonstrate the central influence of the domestic environment, with household-level covariates and shared household accounting for nearly one fifth of total variation in hookworm intensity.

The spatial heterogeneity of parasitic infections has gained increased prominence in recent years [31], [32]. Spatial patterns may not always be evident in maps of unadjusted mean infection intensity because of sampling variation caused by, for example, socio-demographic factors and aggregation of parasite egg counts [33]. The methods adopted here to assess spatial structure have the particular advantage of taking into account the highly skewed distribution of parasite counts, whilst adjusting for covariates [34], [35]. Similar ranges of hookworm spatial clustering have also been reported in Brazil [11] and are consistent with household studies of urinary schistosomiasis [36] and malaria [37]. In contrast, studies of hookworm infection in Cote d'Ivoire [38], [39] and Kenya [40] saw no evidence of household- or school-level spatial clustering at this scale. Differences between sites may be explained by the relative importance of domestic, peri-domestic and public domains in defining contact with infectious stages [41]. For example, previous studies have suggested exposure to infective hookworm larvae commonly occurs outside the household (for example in agricultural areas, schools or defined defecation sites [42]), although our results suggest that in this community transmission occurs within or close to households, consistent with the significant household clustering observed. Residual spatial clustering of hookworm infection in this community may be attributable to small-scale variation in unmeasured environmental factors, such as soil type or vegetation cover [10], [43].

Previous quantitative genetic analysis of helminth infection within human populations suggests roles for both extrinsic (domestic environmental) and intrinsic (genetic) factors in clustering of intestinal nematode infections within households [12], [14], [24], [25], [44], [45]. To date, the only study to investigate heritability in an African setting, conducted in Zimbabwe, suggested heritability of hookworm infection intensity was 0.37, although this study failed to account for household effects, limiting interpretation of findings. Our analysis suggested that, after accounting for household effects and covariates, heritability in this Ugandan population is relatively modest at 0.11, comparable to findings from Papua New Guinea [12] but lower those reported by studies conducted in high transmission settings in Brazil (0.20–0.25) (*Bethony*, *unpublished data*)[13]. Heritability is a population-specific measure, and as such direct comparison of genetic contributions between sites can be confounded by both the differing importance of environmental factors and by variation in genetic polymorphisms of the pathogen/host [46], [47]. Contributions of genetic and household effects may also be influenced by sample size and pedigree and household structures, although studies do suggest that the impact of pedigree structure on precision and accuracy of heritability estimates may be minimal for purely additive genetic effects [48]. It is conceivable however that host genes may play a more important role in higher transmission settings. Alternatively, different immune responses, and thus genes, may influence initial versus re-infection, intensity [49], [50] resulting in lower heritability estimates in this post-treatment setting. Alternatively, since previous treatment could not be measured accurately and instead relied upon participants reporting use of anthelmintic medication, this may have reduced heritability by adding to unexplained variation.

Individual and household risk factors for hookworm infection have been explored extensively, and our findings are in keeping with the literature (see review by Brooker et al (2004)[51], also [52]–[57]). Reassuringly, whilst the two analysis strategies looked at different outcomes (untransformed and log-transformed count data) the significance and magnitude of associations were very similar for each approach, confirming the validity of the findings. Although studies frequently observe higher burdens of hookworm in males than females [1], [6], [51], [58], we could find no evidence of a sex difference here, suggesting an absence of sex-related differences in exposure for this population. Absence of latrine facilities was not associated with hookworm infection risk in this community, although this may be indicative of the relatively uniform distribution of poor quality uncovered pit latrines throughout the community [52], [53]. Similarly, whilst relative socioeconomic group *per se* was not associated with hookworm infection, other indirect measures of household socio-economic status (household member with a formal income, education level of primary carer and household head, walking barefoot when outside the home) were risk factors. Socio-economic status is likely to influence exposure to hookworm infection risk via a number of mechanisms, which may include poor hygienic behaviour and health knowledge [59]–[62].

It is worth noting that for practical reasons we rely on egg counts as an indirect measure of worm burden, and whilst the relationship between egg production and worm burden in hookworm infection is approximately linear, faecal egg counts are known to fluctuate daily, limiting Kato-Katz sensitivity and increasing measurement error [63]. This is reflected by our observation that mean infection intensities were higher for participants who provided two stool samples, suggesting that infections were missed in those that brought only one. As such, we may have underestimated sources of heterogeneity. Furthermore, biases inherent in using faecal egg counts as proxy for worm burden may have added to unexplained heterogeneity. However, the only real alternative for quantifying hookworm infection levels is actual worm burden, which is unfeasible to collect for large population-based studies. A further limitation is the inability to incorporate both genetic and spatial clustering into one model, and as such - as relatives are more likely to live close together - this analysis does risk over-estimating the role of each determinant.

In conclusion, this report provides one of the most comprehensive population-level epidemiological analyses of hookworm infection for sub-Saharan Africa. We demonstrate that, despite several rounds of school-based deworming, prevalence and intensity of infection amongst adults remains high in this community. Such observations have potentially important implications for mass deworming programmes, which currently target only school-aged children. There are currently few data quantifying the benefits of deworming in non-pregnant adult populations, but results presented here and elsewhere [28], [64], [65] suggest that extension of deworming efforts to adult populations may prove a valuable intervention. The feasibility of such an approach is demonstrated by ongoing lymphatic filariasis control, which includes co-administration of ivermectin and albendazole to all (non-pregnant) individuals aged above 4 years and represent the largest community-based helminth control programmes in Africa [66], [67]. From a scientific perspective, the use of complementary spatial and genetic epidemiological approaches has allowed us to update our understanding of the ecology of infection, and has emphasised the continuing importance of household and peri-domestic factors, as well as host genetic relatedness, in influencing infection patterns even after the implementation of control measures.

## Supporting Information

Checklist S1 STROBE checklist.
(0.09 MB DOC)
